# Association of smoking cessation after atrial fibrillation diagnosis on the risk of cardiovascular disease: a cohort study of South Korean men

**DOI:** 10.1186/s12889-020-8275-y

**Published:** 2020-02-03

**Authors:** Seulggie Choi, Jooyoung Chang, Kyuwoong Kim, Sung Min Kim, Hye-Yeon Koo, Mi Hee Cho, In Young Cho, Hyejin Lee, Joung Sik Son, Sang Min Park, Kiheon Lee

**Affiliations:** 1 Department of Biomedical Sciences, Seoul National University Graduate School, Seoul, South Korea; 2 Department of Family Medicine, Seoul National University Bundang Hospital, 82 Gumi-ro, 173 beon-gil, Bundang-gu, Seongnam, South Korea; 3 Department of Family Medicine, Samsung C&T Medical Center, Seoul, South Korea; 4 Department of Family Medicine, Seoul National University Hospital, Seoul, South Korea

**Keywords:** Cohort analysis, Quitting smoking, Atrial fibrillation, Cardiovascular disease

## Abstract

**Background:**

While smoking elevates the risk for cardiovascular disease (CVD) among atrial fibrillation (AF) patients, whether smoking cessation after AF diagnosis actually leads to reduced CVD risk is unclear. We aimed to determine the association of smoking cessation after AF diagnosis with subsequent CVD Risk among South Korean men.

**Methods:**

This retrospective cohort study included 2372 newly diagnosed AF male patients during 2003–2012 from the Korean National Health Insurance Service database. Self-reported smoking status within 2 years before and after diagnosis date were determined, after which the participants were divided into continual smokers, quitters (smokers who quit after AF diagnosis), sustained-ex smokers (those who quit prior to AF diagnosis), and never smokers. Participants were followed up from 2 years after AF diagnosis until 31 December 2015 for CVD. Cox proportional hazards regression was used to determine the adjusted hazard ratios (aHRs) and 95% confidence interval (CIs) for CVD according to the change in smoking habits before and after AF diagnosis.

**Results:**

The mean (standard deviation, minimum-maximum) age of the study subjects was 62.5 (8.6, 41–89) years. Among AF patients, quitters had 35% reduced risk (aHR 0.65, 95% CI 0.44–0.97) and never smokers had 32% reduced risk (aHR 0.68, 95% CI 0.52–0.90) for CVD compared to continual smokers (*p* for trend 0.020). Similarly, compared to continual smokers, quitters had 41% risk-reduction (aHR 0.59, 95% CI 0.35–0.99) and never smokers 34% risk-reduction (aHR 0.66, 95% CI 0.46–0.93) for total stroke (*p* for trend 0.047). Quitters had 50% reduction (aHR 0.50, 95% CI 0.27–0.94), sustained ex-smokers had 36% reduction (aHR 0.64, 95% CI 0.42–0.99), and never smokers had 39% reduction (aHR 0.61, 95% CI 0.41–0.91) in ischemic stroke risk (*p* for trend 0.047). The risk-reducing effect of quitting on CVD risk tended to be preserved regardless of aspirin or warfarin use.

**Conclusions:**

Smoking cessation after AF diagnosis was associated with reduced CVD, total stroke, and ischemic stroke risk.

## Background

The estimated global prevalence of atrial fibrillation (AF) patients was 33.5 million patients in 2010 [1]. Furthermore, approximately one-fourth of middle aged adults are expected to develop AF [2, 3], with an annual incidence rate of 120,000 to 215,000 patients in the European Union alone [4]. AF patients are at two-fold increased risk of mortality [5], in large part due to the elevated risk for cardiovascular disease (CVD). For example, it has been estimated that 20–30% of ischemic stroke patients have AF [6]. While many advances in AF management such as anticoagulation therapy has been made [7], substantial morbidity still remains [8]. Therefore, identifying and managing modifiable risk factors for CVD among AF patients are of importance from clinical and public health perspectives.

Smoking is one of the most common health behavior factors related to CVD, and the effect of smoking on CVD among AF patients has previously been studied [9, 10]. Multiple longitudinal studies from developed countries such as the United Kingdom and Netherlands have shown that there was a higher risk for CVD among smokers compared to never smokers [9, 10]. However, such studies only determined smoking status at one point in time and thus could not elucidate whether the change in smoking habit over time alters the risk of CVD among AF patients [9, 10]. Moreover, there is a relative lack of evidence among populations residing in Asian countries. While recent AF management guidelines suggest behavioral modification including smoking cessation [11], there is currently a lack of evidence on whether quitting after AF diagnosis actually leads to reduced CVD risk among smokers.

Therefore, in this longitudinal study using the Korean National Health Insurance Service (NHIS) database, we investigated the association of smoking habit change on the risk of CVD among newly diagnosed AF male patients.

## Methods

### Study design and setting

The study subjects were derived from the National Health Insurance Service – Health Screening Cohort (NHIS-HEALS, NHIS-2018-2-118). In South Korea, the NHIS provides mandatory health insurance covering nearly all forms of health services for all Korean citizens, resulting in an enrollment rate of approximately 98% [12]. Furthermore, all enrollees aged 40 years or older are required to undergo biannual health screening examinations that include a self-reported questionnaire on health behavior, body measurements including height, weight, and blood pressure, as well as blood and urine laboratory exams. Based on this claims database, the NHIS provides a part of their data for research purposes, which include information on sociodemographics, inpatient and outpatient hospital use, medication prescriptions, and results from health screening examinations [13]. The NHIS database has previously been used for a wide range of epidemiological studies, and its validity is described in detail elsewhere [12, 14].

### Study subjects and period

**Fig. 1:**
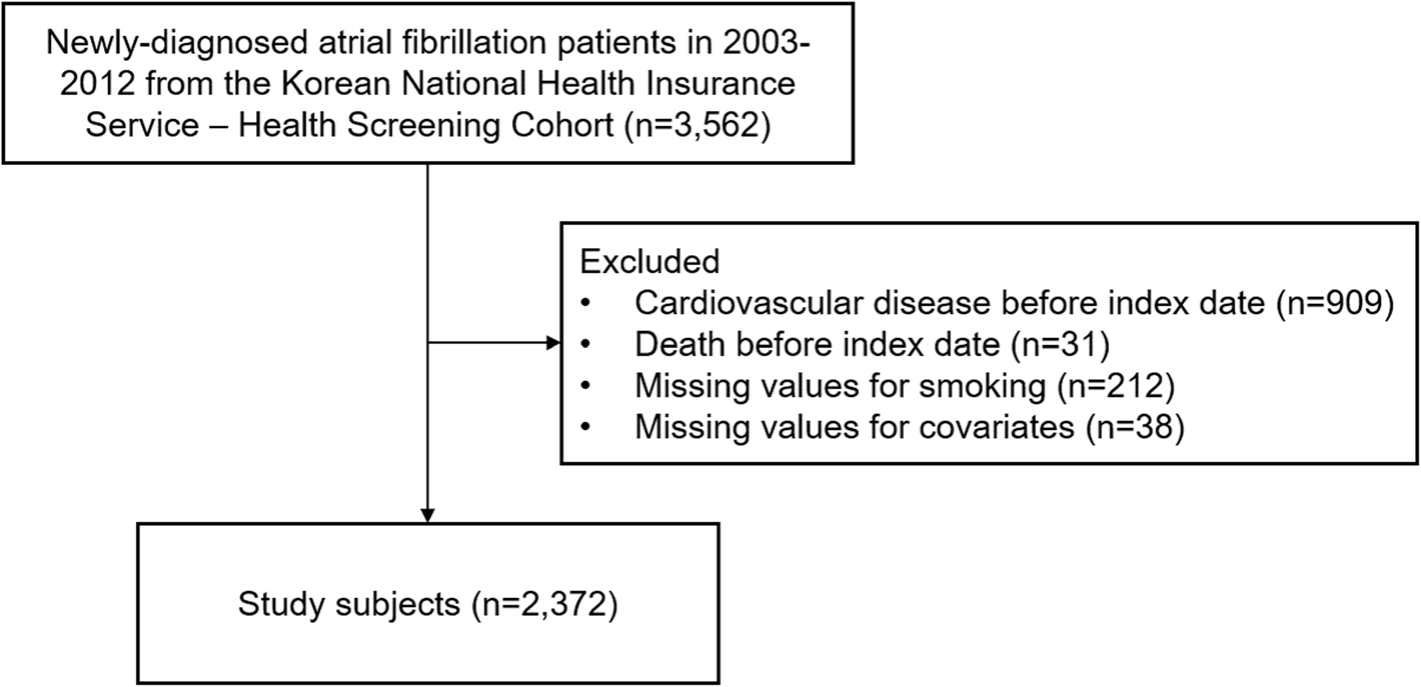
Title Flow diagram of the study subjects

Among 3562 newly diagnosed AF male patients (age 41–89 years) during 2003–2012, we excluded 909 patients who were diagnosed with CVD before the index date. Furthermore, 31 patients who died before the index date were also excluded. Finally, 212 and 38 patients with missing information on smoking status and covariates were excluded, respectively. The final study subjects consisted of 2372 AF patients (Fig. 1). Smoking status was determined within 2 years before and after AF diagnosis to determine smoking habit change. Starting from the index date of 2 years after AF diagnosis date, participants were followed-up until 2015 for CVD.

### Key variables

AF was defined as either a hospitalization or two or more outpatient department visits under the diagnosis of AF [15]. Upon hospital use, the NHIS requires physicians to enter a diagnosis for all patients using the International Classification of Diseases, Tenth Revision (ICD-10) codes. The ICD-10 codes used for AF diagnosis were I48.0-I48.4, and I48.9 [16] Similarly, CVD was defined upon two or more days of admission or death due to coronary heart disease (ICD-10 codes I20-I25) or total stroke (ICD-10 codes I60-I69) [17]. Acute myocardial infarction (ICD-10 code I21) was also separately detected. Under total stroke, ischemic stroke (ICD-10 code I63) and hemorrhagic stroke (ICD-10 codes I61-I62) events were also determined.

Smoking status was determined by a self-reported questionnaire during health screening examinations within 2 years prior to (first health examination) and 2 years after (second health examination) AF diagnosis. The self-reported questionnaire requires the participant to choose between being a current smoker, past smoker, or never smoker according to his current smoking status at the time of the examination. Based on the smoking status from before and after AF diagnosis, all participants were grouped into either continual smokers, quitters, sustained ex-smokers, and never smokers. Continual smokers were those who were current smokers both before and after AF diagnosis. Quitters were participants who were current smokers before AF diagnosis that became quitters after AF diagnosis. Sustained ex-smokers were those who were quitters both before and after AF diagnosis. Finally, never smokers were participants who were never smokers both before and after AF diagnosis.

The considered covariates included age (continuous, years), household income (categorical, 1st, 2nd, 3rd, and 4th quartiles), alcohol consumption (categorical, 0, 0–1, 1–2, 3–4, and ≥ 5 times per week), physical exercise (categorical, 0, 1–2, 3–4, 5–6, and 7 times per week), body mass index (continuous, kg/m^2^), systolic blood pressure (continuous, mmHg), fasting serum glucose (continuous, mg/dL), total cholesterol (continuous, mg/dL), Charlson comorbidity index (categorical, ≤1, 2, 3, and ≥ 4), aspirin use (categorical, yes and no), warfarin use (categorical, yes and no), and index year. Household income was derived from the insurance premium and body mass index was calculated by dividing the height in meters by weight in kilograms squared. The algorithm for calculating Charlson comorbidity index from claims data was derived from a previous study [18].

### Statistical analysis

Multivariate Cox proportional hazards regression was used to calculate the adjusted hazard ratios (aHRs) and 95% confidence intervals (CIs) for CVD according to smoking habit change after adjustments for all covariates mentioned above. For all analyses, continual smokers were the reference group in order to assess the risk of quitting compared to continually smoking, which is in line with previous studies that also determined changes in smoking habit as the primary exposure [14, 19, 20]. All participants were followed-up starting from the index until the date of CVD, death, or 31 December 2015, whichever came first. Furthermore, a stratified analysis for the association of smoking habit change on CVD according to subgroups of aspirin and warfarin use was conducted. Statistical significance was defined as a *p* value of < 0.05 in a two sided manner. All data analyses were conducted using SAS version 9.4 (SAS Institute Inc).

## Results

Table 1 depicts the descriptive characteristics of the study subjects. A total of 2372 AF patients were followed up for a mean (minimum-maximum) duration of 5.0 (0.1–10.8) years. The number of patients who were continual smokers, quitters, sustained ex-smokers, and never smokers were 475, 251, 779, and 867, respectively. The mean (standard deviation) age for continual smokers, quitters, sustained ex-smokers, and never smokers were 59.9 (9.4), 60.7 (9.6), 62.9 (9.9), and 64.1 (9.6) years, respectively. Compared to continual smokers, quitters tended to be older, have higher household income, consume less alcohol, have lower comorbidities, and use warfarin more.

**Table 1:** Basal characteristics of the study subjects according to groups of changes in smoking habit

	Continual smokers	Quitters	Sustained ex-smokers	Never smokers
Number of people	475	251	779	867
Age, years, mean (SD)	59.9 (9.4)	60.7 (9.6)	62.9 (9.9)	64.1 (9.6)
Household income, quartiles, N (%)				
1st (highest)	160 (33.7)	101 (40.2)	365 (46.9)	377 (43.5)
2nd	155 (32.6)	65 (25.9)	197 (25.3)	238 (27.5)
3rd	94 (19.8)	55 (21.9)	133 (17.1)	145 (16.7)
4th (lowest)	66 (13.9)	30 (12.0)	84 (10.8)	107 (12.3)
Alcohol consumption, times per week, N (%)				
0	146 (30.7)	136 (54.2)	356 (45.7)	513 (59.2)
0–1	91 (19.2)	42 (16.7)	141 (18.1)	143 (16.5)
1–2	116 (24.4)	43 (17.1)	122 (15.7)	116 (13.4)
3–4	60 (12.6)	21 (8.4)	95 (12.2)	62 (26.1)
≥ 5	62 (13.1)	9 (3.6)	65 (8.3)	33 (3.8)
Physical exercise, times per week, N (%)				
0	227 (47.8)	133 (53.0)	340 (43.7)	407 (46.9)
1–2	145 (30.5)	53 (21.1)	219 (28.1)	206 (23.8)
3–4	56 (11.8)	30 (12.0)	121 (15.5)	117 (13.5)
5–6	18 (3.8)	13 (5.2)	56 (7.2)	54 (6.2)
7	29 (6.1)	22 (8.8)	43 (5.5)	83 (9.6)
Body mass index, kg/m^2^, mean (SD)	23.8 (3.0)	23.9 (3.1)	24.4 (2.9)	24.2 (2.8)
Systolic blood pressure, mmHg, mean (SD)	125.1 (16.5)	124.5 (16.1)	125.8 (15.7)	126.6 (15.6)
Fasting serum glucose, mg/dL, mean (SD)	104.0 (32.3)	103.1 (27.5)	104.8 (28.2)	102.8 (27.5)
Total cholesterol, mg/dL, mean (SD)	188.7 (36.1)	192.2 (38.1)	185.6 (35.2)	182.3 (35.8)
Charlson comorbidity index, N (%)				
≤ 1	185 (39.0)	84 (33.5)	281 (36.1)	333 (38.4)
2	106 (22.3)	49 (19.5)	163 (20.9)	172 (19.8)
3	80 (14.7)	37 (14.7)	128 (16.4)	151 (17.4)
≥ 4	114 (24.0)	81 (32.3)	207 (26.6)	211 (24.3)
Aspirin use, N (%)	277 (58.3)	151 (60.2)	501 (64.3)	558 (64.4)
Warfarin use, N (%)	79 (16.6)	58 (23.1)	183 (23.5)	232 (26.8)

*Acronyms*: *SD* standard deviation

The association of smoking habit change on CVD among newly diagnosed AF male patients is shown in Table 2. Compared to continual smokers, quitters had 35% reduced risk (aHR 0.65, 95% CI 0.44–0.97) and never smokers had 32% reduced risk (aHR 0.68, 95% CI 0.52–0.90) for CVD (*p* for trend 0.020). Similarly, quitters had 41% risk-reduction (aHR 0.59, 95% CI 0.35–0.99) and never smokers had 34% risk-reduction (aHR 0.66, 95% CI 0.46–0.93) for total stroke risk compared to continual smokers (*p* for trend 0.047). Finally, for ischemic stroke, quitters had 50% reduction (aHR 0.50, 95% CI 0.27–0.94), sustained ex-smokers had 36% reduction (aHR 0.64, 95% CI 0.42–0.99), and never smokers had 38% reduction (aHR 0.62, 95% CI 0.41–0.91) in risk compared to continual smokers (*p* for trend 0.047). There was a significant trend of risk reduction for CVD, total stroke, and ischemic stroke upon decreasing levels of tobacco consumption from continual smokers to never smokers.

**Table 2:** Hazard ratios for cardiovascular disease according to the change in smoking habit among atrial fibrillation male patients

	Continual smokers	Quitters	Sustained ex-smokers	Never smokers	*p* for trend
Cardiovascular disease					
Events	93	35	110	151	
Person-years	2407	1312	3382	4666	
aHR (95% CI)^a^	1.00 (reference)	0.65 (0.44–0.97)	0.76 (0.57–1.02)	0.68 (0.52–0.90)	0.020
Total stroke					
Events	60	20	65	92	
Person-years	2541	1374	3512	4871	
aHR (95% CI)^a^	1.00 (reference)	0.59 (0.35–0.99)	0.72 (0.50–1.04)	0.66 (0.46–0.93)	0.047
Ischemic stroke					
Events	46	13	44	64	
Person-years	2589	1395	3568	4942	
aHR (95% CI)^a^	1.00 (reference)	0.50 (0.27–0.94)	0.64 (0.42–0.99)	0.61 (0.41–0.91)	0.047
Hemorrhagic stroke					
Events	6	3	8	11	
Person-years	2687	1414	3659	5117	
aHR (95% CI)^a^	1.00 (reference)	0.96 (0.23–4.00)	1.00 (0.34–3.00)	0.88 (0.31–2.54)	0.805
Coronary heart disease					
Events	44	19	53	79	
Person-years	2545	1347	3527	4904	
aHR (95% CI)^a^	1.00 (reference)	0.75 (0.43–1.29)	0.77 (0.51–1.17)	0.74 (0.50–1.10)	0.189
Acute myocardial infarction					
Events	12	2	4	20	
Person-years	2682	1411	3674	5136	
aHR (95% CI)^a^	1.00 (reference)	0.27 (0.06–1.23)	0.21 (0.06–0.67)	0.67 (0.31–1.45)	0.530

*Acronyms*: *aHR* adjusted hazard ratio, *CI* confidence interval

^a^Hazard ratios calculated by Cox proportional hazards regression analysis after adjustments for age, household income, alcohol consumption, physical exercise, body mass index, systolic blood pressure, fasting serum glucose, total cholesterol, Charlson comorbidity index, aspirin use, warfarin use, and index year

Table 3 shows the stratified analysis for the association of smoking habit change on CVD according to subgroups of aspirin and warfarin use. Sustained ex-smokers had 45% reduced risk (aHR 0.55, 95% CI 0.34–0.90) and never smokers had 45% reduced risk (aHR 0.55, 95% CI 0.35–0.86) for CVD compared to continual smokers among aspirin non-users. Compared to continual smokers, sustained ex-smokers had 29% reduced risk (aHR 0.71, 95% CI 0.51–0.98) and never smokers had 27% reduced risk (aHR 0.73, 95% CI 0.54–0.99) for CVD among warfarin non-users. Never smokers among warfarin users had 48% reduced risk for CVD compared to continual smokers (aHR 0.52, 95% CI 0.28–0.97). Compared to continual smokers, sustained ex-smokers had 56% reduced risk for total stroke among aspirin non-users (aHR 0.44, 95% CI 0.21–0.91). Never smokers had 54% reduced risk for coronary heart disease among aspirin non-users (aHR 0.46, 95% CI 0.25–0.84, *p* for trend 0.018). Finally, never smokers had 59% reduced risk for coronary heart disease compared to continual smokers among warfarin users (aHR 0.41, 95% CI 0.20–0.86).

**Table 3:** Stratified analysis for the association of smoking habit change on cardiovascular disease according to subgroups of aspirin or warfarin use

		Adjusted hazard ratio (95% confidence interval)^a^				
		Continual smokers	Quitters	Sustained ex-smokers	Never smokers	*p* for trend
Cardiovascular disease	Aspirin use					
	No	1.00 (reference)	0.59 (0.31–1.13)	0.55 (0.34–0.90)	0.55 (0.35–0.86)	0.013
	Yes	1.00 (reference)	0.70 (0.42–1.16)	0.94 (0.65–1.35)	0.80 (0.56–1.14)	0.361
	Warfarin use					
	No	1.00 (reference)	0.66 (0.43–1.03)	0.71 (0.51–0.98)	0.73 (0.54–0.99)	0.073
	Yes	1.00 (reference)	0.45 (0.18–1.13)	0.85 (0.45–1.61)	0.52 (0.28–0.97)	0.088
Total stroke	Aspirin use					
	No	1.00 (reference)	0.63 (0.26–1.54)	0.44 (0.21–0.91)	0.71 (0.38–1.31)	0.269
	Yes	1.00 (reference)	0.83 (0.40–1.69)	1.04 (0.61–1.77)	0.79 (0.47–1.33)	0.423
	Warfarin use					
	No	1.00 (reference)	0.70 (0.38–1.29)	0.74 (0.46–1.16)	0.78 (0.51–1.20)	0.343
	Yes	1.00 (reference)	0.64 (0.17–2.46)	0.85 (0.31–2.35)	0.54 (0.20–1.49)	0.255
Coronary heart disease	Aspirin use					
	No	1.00 (reference)	0.53 (0.22–1.29)	0.60 (0.31–1.14)	0.46 (0.25–0.84)	0.018
	Yes	1.00 (reference)	0.62 (0.32–1.19)	0.87 (0.55–1.38)	0.83 (0.54–1.29)	0.661
	Warfarin use					
	No	1.00 (reference)	0.64 (0.36–1.15)	0.68 (0.44–1.04)	0.74 (0.50–1.10)	0.197
	Yes	1.00 (reference)	0.32 (0.10–1.03)	0.67 (0.32–1.42)	0.41 (0.20–0.86)	0.053

^a^Hazard ratios calculated by Cox proportional hazards regression analysis after adjustments for age, household income, alcohol consumption, physical exercise, body mass index, systolic blood pressure, fasting serum glucose, total cholesterol, Charlson comorbidity index, aspirin use, warfarin use, and index year

## Discussion

In this longitudinal study of 2372 newly diagnosed AF male patients, we have shown that smoking cessation after AF diagnosis was associated with reduced CVD, total stroke, and ischemic stroke risk. This association of CVD risk-reduction upon smoking cessation did not appear to alter significantly due to aspirin or warfarin use. To our knowledge, this is the first study to show that quitting after diagnosis of AF among smokers may benefit from reduced risk of CVD.

While we could not find any studies on the association of smoking habit change on CVD risk among AF patients, multiple previous studies have investigated the effect of smoking measured at one point in time on CVD risk among AF patients. In a study that investigated risk factors for stroke among AF patients, the risk of stroke was not elevated among current smokers compared to never smokers (*p* value 0.08) [21]. However, another study by Lip and colleagues showed that smoking was associated with higher thromboembolic event risk (aHR 2.10, 95% CI 1.38–3.18) [10]. Similarly, a recent study by Albertsen and colleagues have shown that smoking was associated with higher risk of thromboembolism [9]. Current male smokers with ≤25 g/d (aHR 1.66, 95% CI 1.30–2.12) and > 25 g/day (aHR 2.17, 95% CI 1.59–2.95) of tobacco consumption had elevated risk for thromboembolism compared to never smokers [9]. Our study adds to the results from previous investigations by showing that smoking cessation was associated with reduced CVD risk among AF patients who initially smoked.

Multiple pathophysiological mechanisms may be at play in the risk-increasing effect of smoking on CVD. Smoking has been shown to reduce vasodilatory function in both human [22] and animal [23] models, partly by decreasing nitric oxide availability [24]. Furthermore, smoking also may increase peripheral leukocyte count [25], C-reactive protein levels [26], and interleukin-6 [27], all of which are associated with inflammation. Both vasomotor dysfunction and increases in systemic inflammatory states could lead to higher risk for atherosclerosis, which in turn could result in CVD [28]. Additionally, smoking has previously been shown to elevate low-density lipoprotein and triglyceride levels, while reducing high-density lipoprotein (HDL) [29]. This may be achieved in part by increasing lipid oxidation [30] and inhibiting lecithin cholesterol acyl-transferase activity [31], an important enzyme that helps maintain HDL levels [32].

Another primary mechanism of smoking on increasing CVD risk is by promoting a hypercoagulable state by inducing platelet dysfunction [33] and alteration of antithrombotic factors [34]. Therefore, whether antithrombotic therapy such as aspirin and warfarin medication, which are frequently prescribed in AF patients, alters the risk-reducing effect of quitting on CVD is of clinical importance. We have thus conducted a stratified analysis on the association between smoking habit change and CVD according to subgroups of aspirin and warfarin use. Despite the lack of significance likely due to the reduction in statistical power, the risk of CVD, total stroke, and coronary heart disease upon smoking cessation did not appear to alter significantly according to aspirin or warfarin use. Consequently, the antithrombotic effect of aspirin and warfarin does not appear to attenuate the risk-reducing effect of quitting on CVD risk among AF patients.

Several limitations must be considered when interpreting the results from our study. First, as smoking status was determined using a self-reported questionnaire, there could have been an underestimation of current smokers. This may be particularly true for past smokers, which could partly explain the lack of significant CVD risk-reduction among sustained ex-smokers since this group may actually contain current smokers who reported having quit during the health screening. Therefore, future studies that use a more reliable method of smoking status, such as urine cotinine levels, are needed to validate the findings of this study. Second, we did not take into account additional changes in smoking habit beyond the index date, due to the low number of participants who underwent health examinations after the index date. Since many smokers are unable to maintain quitting beyond one year [35], a number of patients grouped as quitters in our study may actually have become current smokers after the index date (i.e. relapsers), possibly leading to an underestimation of the risk-reducing effect of quitting on CVD. Moreover, participants who were never smokers may have initiated smoking after the index date. Future studies that take into account multiple smoking status changes are needed to validate our findings.

Third, the study subjects consisted of male patients who underwent health screening examinations, which may be associated with certain sociodemographic tendencies. Although we attempted to take this into account by adjusting for a number of sociodemographic and health behavior factors, future studies with study subjects that include women and a more general population are needed. Fourth, we could not take into account the severity of AF due to the lack of data on medical chart records, which could be an important confounder in our study. Although we attempted to take this into account by limiting the study subjects to newly diagnosed patients, future studies that use health records to determine AF severity would be beneficial. Finally, we could not accurately assess the association of smoking cessation after AF diagnosis on the risk of acute myocardial infarction due to the lack of enough cases (2 cases of acute myocardial infarction among quitters) despite the observed significant aHR value. Future studies with a greater number of study subjects and acute myocardial infarction cases are needed.

Despite these limitations, our study has a number of strengths. First, we determined smoking status before and after AF diagnosis, which enabled us to investigate the risk of CVD according to the change in smoking habit. Second, the study subjects consisted of AF patients, a group that has not previously been studied on the association between smoking habit change and CVD risk. Third, the extensive list of covariates spanning from sociodemographic factors to health behavior and health status allowed us to enhance the reliability of our findings. Finally, the longitudinal design gives support to the cause-and-effect relationship of smoking habit change having effects on CVD risk.

## Conclusions

Quitting was associated with decreased CVD risk among newly diagnosed AF male patients in South Korea. The beneficial effect of smoking cessation on CVD risk did not appear to be altered significantly by aspirin or warfarin use. Future prospective, intervention studies are needed to determine whether smokers who quit after AF diagnosis may benefit from reduced CVD risk.

## Data Availability

The data that support the findings of this study are available from the Korean NHIS but restrictions apply to the availability of these data, which were used under license for the current study, and so are not publicly available. Data are however available from the authors upon reasonable request and with permission of the Korean NHIS.

## Abbreviations

AF: Atrial fibrillation
aHR: Adjusted hazard ratio
CI: Confidence interval
CVD: Cardiovascular disease
NHIS-HEALS: National Health Insurance Service – Health Screening Cohort

## References

[1] Chugh SS, Havmoeller R, Narayanan K, Singh D, Rienstra M, Benjamin EJ, Gillum RF, Kim YH, McAnulty JH, Zheng ZJ (2014). Worldwide epidemiology of atrial fibrillation: a global burden of disease 2010 study. Circulation.

[2] Heeringa J, van der Kuip DA, Hofman A, Kors JA, van Herpen G, Stricker BH, Stijnen T, Lip GY, Witteman JC (2006). Prevalence, incidence and lifetime risk of atrial fibrillation: the Rotterdam study. Eur Heart J.

[3] Go AS, Hylek EM, Phillips KA, Chang Y, Henault LE, Selby JV, Singer DE (2001). Prevalence of diagnosed atrial fibrillation in adults: national implications for rhythm management and stroke prevention: the AnTicoagulation and risk factors in atrial fibrillation (ATRIA) study. JAMA.

[4] Zoni-Berisso M, Lercari F, Carazza T, Domenicucci S (2014). Epidemiology of atrial fibrillation: European perspective. Clin Epidemiol.

[5] Andersson T, Magnuson A, Bryngelsson IL, Frobert O, Henriksson KM, Edvardsson N, Poci D (2013). All-cause mortality in 272,186 patients hospitalized with incident atrial fibrillation 1995-2008: a Swedish nationwide long-term case-control study. Eur Heart J.

[6] Kishore A, Vail A, Majid A, Dawson J, Lees KR, Tyrrell PJ, Smith CJ (2014). Detection of atrial fibrillation after ischemic stroke or transient ischemic attack: a systematic review and meta-analysis. Stroke.

[7] Xu J, Luc JG, Phan K (2016). Atrial fibrillation: review of current treatment strategies. J Thorac Dis.

[8] Alonso A, Norby FL (2016). Predicting atrial fibrillation and its complications. Circ J.

[9] Albertsen IE, Rasmussen LH, Lane DA, Overvad TF, Skjoth F, Overvad K, Lip GY, Larsen TB (2014). The impact of smoking on thromboembolism and mortality in patients with incident atrial fibrillation: insights from the Danish diet, Cancer, and health study. Chest.

[10] Lip GY, Frison L, Halperin JL, Lane DA (2010). Identifying patients at high risk for stroke despite anticoagulation: a comparison of contemporary stroke risk stratification schemes in an anticoagulated atrial fibrillation cohort. Stroke.

[11] Kirchhof P, Benussi S, Kotecha D, Ahlsson A, Atar D, Casadei B, Castella M, Diener HC, Heidbuchel H, Hendriks J (2016). 2016 ESC guidelines for the management of atrial fibrillation developed in collaboration with EACTS. Eur Heart J.

[12] Cheol Seong S, Kim YY, Khang YH, Heon Park J, Kang HJ, Lee H, Do CH, Song JS, Hyon Bang J, Ha S (2017). Data resource profile: the National Health Information Database of the National Health Insurance Service in South Korea. Int J Epidemiol.

[13] Seong SC, Kim YY, Park SK, Khang YH, Kim HC, Park JH, Kang HJ, Do CH, Song JS, Lee EJ (2017). Cohort profile: the National Health Insurance Service-National Health Screening Cohort (NHIS-HEALS) in Korea. BMJ Open.

[14] Kim K, Park SM, Lee K (2018). Weight gain after smoking cessation does not modify its protective effect on myocardial infarction and stroke: evidence from a cohort study of men. Eur Heart J.

[15] Son MK, Lim NK, Kim HW, Park HY (2017). Risk of ischemic stroke after atrial fibrillation diagnosis: a national sample cohort. PLoS One.

[16] Kim TH, Yang PS, Uhm JS, Kim JY, Pak HN, Lee MH, Joung B, Lip GYH (2017). CHA2DS2-VASc score (congestive heart failure, hypertension, age >/=75 [doubled], diabetes mellitus, prior stroke or transient ischemic attack [doubled], vascular disease, age 65-74, female) for stroke in Asian patients with atrial fibrillation: a Korean Nationwide sample cohort study. Stroke.

[17] Mozaffarian D, Benjamin EJ, Go AS, Arnett DK, Blaha MJ, Cushman M, Das SR, de Ferranti S, Despres JP, Writing Group M (2016). Heart disease and stroke Statistics-2016 update: a report from the American Heart Association. Circulation.

[18] Quan H, Sundararajan V, Halfon P, Fong A, Burnand B, Luthi JC, Saunders LD, Beck CA, Feasby TE, Ghali WA (2005). Coding algorithms for defining comorbidities in ICD-9-CM and ICD-10 administrative data. Med Care.

[19] Choi S, Kim K, Chang J, Kim SM, Koo HY, Jun JH, Cho MH, Lee K, Park SM (2017). Effect of post-cessation hyperglycemia on cardiovascular disease and mortality among middle-aged men: an eight-year longitudinal study. Sci Rep.

[20] Clair C, Rigotti NA, Porneala B, Fox CS, D'Agostino RB, Pencina MJ, Meigs JB (2013). Association of smoking cessation and weight change with cardiovascular disease among adults with and without diabetes. JAMA.

[21] Atrial Fibrillation Investigators. Risk factors for stroke and efficacy of antithrombotic therapy in atrial fibrillation. Analysis of pooled data from five randomized controlled trials. Arch Intern Med. 1994;154(13):1449–57. https://www.ncbi.nlm.nih.gov/pubmed/8018000. 8018000

[22] Celermajer DS, Sorensen KE, Georgakopoulos D, Bull C, Thomas O, Robinson J, Deanfield JE (1993). Cigarette smoking is associated with dose-related and potentially reversible impairment of endothelium-dependent dilation in healthy young adults. Circulation.

[23] Mayhan WG, Sharpe GM (1996). Effect of cigarette smoke extract on arteriolar dilatation in vivo. J Appl Physiol (1985).

[24] Ota Y, Kugiyama K, Sugiyama S, Ohgushi M, Matsumura T, Doi H, Ogata N, Oka H, Yasue H (1997). Impairment of endothelium-dependent relaxation of rabbit aortas by cigarette smoke extract--role of free radicals and attenuation by captopril. Atherosclerosis.

[25] Smith CJ, Fischer TH (2001). Particulate and vapor phase constituents of cigarette mainstream smoke and risk of myocardial infarction. Atherosclerosis.

[26] Tracy RP, Psaty BM, Macy E, Bovill EG, Cushman M, Cornell ES, Kuller LH (1997). Lifetime smoking exposure affects the association of C-reactive protein with cardiovascular disease risk factors and subclinical disease in healthy elderly subjects. Arterioscler Thromb Vasc Biol.

[27] Bermudez EA, Rifai N, Buring JE, Manson JE, Ridker PM (2002). Relation between markers of systemic vascular inflammation and smoking in women. Am J Cardiol.

[28] Ambrose JA, Barua RS (2004). The pathophysiology of cigarette smoking and cardiovascular disease: an update. J Am Coll Cardiol.

[29] Craig WY, Palomaki GE, Haddow JE (1989). Cigarette smoking and serum lipid and lipoprotein concentrations: an analysis of published data. BMJ.

[30] Heitzer T, Yla-Herttuala S, Luoma J, Kurz S, Munzel T, Just H, Olschewski M, Drexler H (1996). Cigarette smoking potentiates endothelial dysfunction of forearm resistance vessels in patients with hypercholesterolemia. Role of oxidized LDL. Circulation.

[31] McCall MR, van den Berg JJ, Kuypers FA, Tribble DL, Krauss RM, Knoff LJ, Forte TM (1994). Modification of LCAT activity and HDL structure. New links between cigarette smoke and coronary heart disease risk. Arterioscler Thromb.

[32] Chelland Campbell S, Moffatt RJ, Stamford BA (2008). Smoking and smoking cessation -- the relationship between cardiovascular disease and lipoprotein metabolism: a review. Atherosclerosis.

[33] Fusegawa Y, Goto S, Handa S, Kawada T, Ando Y (1999). Platelet spontaneous aggregation in platelet-rich plasma is increased in habitual smokers. Thromb Res.

[34] Barua RS, Ambrose JA, Saha DC, Eales-Reynolds LJ (2002). Smoking is associated with altered endothelial-derived fibrinolytic and antithrombotic factors: an in vitro demonstration. Circulation.

[35] Hughes JR, Carpenter MJ (2006). Does smoking reduction increase future cessation and decrease disease risk? A qualitative review. Nicotine Tob Res.

